# Development and Evaluation of Five-in-One Vaccine Microneedle Array Patch for Diphtheria, Tetanus, Pertussis, Hepatitis B, and *Haemophilus influenzae* Type b: Immunological Efficacy and Long-Term Stability

**DOI:** 10.3390/pharmaceutics16121631

**Published:** 2024-12-23

**Authors:** In-Jeong Choi, Hye-Ran Cha, Danbi Kwon, Aram Kang, Ji Seok Kim, Jooyoung Kim, Jeong-Eun Choi, Hyeon Woo Chung, Sunghoon Park, Doo Hee Shim, Tae-Hyun Kim, Seung-Ki Baek, Woon-Sung Na, Jae Myun Lee, Jung-Hwan Park

**Affiliations:** 1 QuadMedicine R&D Centre, QuadMedicine, Inc., Seongnam 13209, Republic of Korea; cij@quadmedicine.com (I.-J.C.); aramkang@quadmedicine.com (A.K.); j.kim@quadmedicine.com (J.K.);; 2 Department of Microbiology and Immunology, Institute for Immunology and Immunological Diseases, Graduate School of Medical Science, Yonsei University College of Medicine, Seoul 03722, Republic of Korea; hrcha@yuhs.ac (H.-R.C.);; 3 LG Chem Ltd., Seoul 07796, Republic of Korea; taehyunk@lgchem.com; 4 Department of Oral Microbiology and Immunology, School of Dentistry, Seoul National University, Seoul 08826, Republic of Korea; 5 Department of BioNano Technology, Gachon University, Seongnam 13120, Republic of Korea

**Keywords:** combination vaccine, microneedle patch, microneedle array, thermal stability, immunological efficacy

## Abstract

**Background and objectives**: The development of a five-in-one vaccine microneedle patch (five-in-one MN patch) aims to address challenges in administering vaccines against Diphtheria (DT), Tetanus (TT), Pertussis (wP), Hepatitis B (HBsAg), and *Haemophilus influenzae* type b (Hib). Combining multiple vaccines into a single patch offers a novel solution to improve vaccine accessibility, stability, and delivery efficiency, particularly in resource-limited settings. **Methods**: The five-in-one MN patch consists of four distinct microneedle arrays: DT and TT vaccines are coated together on one array, while wP, HepB, and Hib vaccines are coated separately on individual arrays. The patch was tested for long-term stability (12 months at 25 °C) and evaluated for immunogenicity in mice and minipigs. Antibody titers were measured using ELISA to compare immune responses between microneedle-based delivery and traditional intramuscular (IM) injection. **Results**: The five-in-one MN patch demonstrated stable antigenicity for up to 12 months at room temperature. In animal studies, the patch induced antibody titers comparable to traditional IM injections for all vaccines. Notably, immunogenic responses to Pertussis and *Haemophilus influenzae* type b vaccines via microneedles were reported for the first time. The patch facilitated the simultaneous yet independent delivery of vaccines, preserving their immunogenicity without interference. **Conclusions**: The five-in-one MN patch represents a significant advancement in vaccine delivery by enabling stable, minimally invasive, and efficient immunization. Its innovative design addresses the critical limitations of combination vaccines and has the potential to enhance vaccine accessibility in low- and middle-income countries. Future studies will focus on optimizing patch application techniques and evaluating broader clinical applicability.

## 1. Introduction

Since 2019, there has been a dramatic increase in the number of unvaccinated children and zero-dose children worldwide due to the COVID-19 pandemic, increased economic crises and conflict, and declining confidence in vaccines. There are staggering inequalities between regions, and while Africa has the highest incidence of zero-dose children, inequalities exist within other regions as well [1].

Reversing the unprecedented rise in zero-dose children requires the development of combination vaccines that are heat-resistant and easy-to-use to ensure life-saving vaccines reach all children. In 2021, the Vaccine Innovation Prioritization Strategy (VIPS) selected microneedle vaccines as a useful vaccination platform to overcome vaccination barriers to ensure equitable access and improved efficacy of vaccines in LMICs and to contribute to global health security [1].

Diphtheria, Tetanus, Hepatitis B (Hep B), Pertussis, and *Haemophilus influenzae* type b (Hib) infections occur frequently in infants and young children, with high rates of morbidity and mortality [2,3,4,5]. Therefore, vaccination against these five diseases is highly recommended for infants and young children, especially in most countries. A mixture of combination vaccines (five-in-one or combination vaccine) for treating these diseases have been produced and are administered intramuscularly by liquid injection [2,6,7,8,9].

Because each vaccine has different biophysical properties and utilizes different manufacturing processes, combination vaccines face various technical challenges. However, the efficacy of a combination vaccine can be reduced due to physical and/or immunological interference between the vaccines. [6,7,10,11,12,13,14,15]. The five-in-one vaccine used in this study consists of recombinant (Hep B), conjugated (Hib), whole-cell (Pertussis), and toxoid (Tetanus and Diphtheria) vaccines. In DTaP-based pentavalent vaccines, studies have reported reduced antibody titers to the *Haemophilus influenzae* type b (Hib) component due to immune interference [16]. This immune interference highlights a critical challenge when developing and optimizing combination vaccines to ensure balanced immunogenicity across all antigens. Besides the issues already mentioned with a combination vaccine, the quantitative analysis of a combination vaccine like a five-in-one vaccine is very challenging because each vaccine requires different methods for measuring antigen quantity and determining antigenicity.

Previous studies have demonstrated that microneedles can deliver various types of vaccines into the skin and induce immunological responses comparable to those induced by intramuscular administration [17,18,19,20,21,22]. Since the vaccine microneedles contain solid vaccine formulation, they can provide improved thermal stability, and they deliver vaccines in a minimally invasive manner that is specifically attributable to the small size of the individual microneedles [22,23,24,25,26,27,28,29,30]. The Vaccine Innovation Prioritization Strategy (VIPS) identifies microneedle array patches (MAPs) as a transformative platform to improve vaccine accessibility, particularly in low- and middle-income countries (LMICs) [31]. Prioritized vaccines include pentavalent, measles–rubella, and HPV, where MAPs offer significant advantages, such as ease of administration and reduced cold chain dependence. Microneedles containing multiple vaccines can be formed by the following procedures: (1) combining multiple vaccines into one solution; (2) concentrating the antigen mixture; (3) preparing a solution containing excipients such as stabilizers and polymers and mixing with the concentrated antigen solution; and (4) forming vaccine microneedles by micro-molding or coating processes. Although vaccine microneedle fabrication methods are well established, microneedles containing multiple vaccines would raise the same problems as a combination vaccine, including reduced efficacy and increased complexity of analysis. Pentavalent vaccines face challenges in resource-limited settings due to cold chain requirements and the need for skilled personnel. Microneedle array patch (MAP) systems address these issues by enabling thermostable, needle-free, and potentially self-administered vaccine delivery. This approach could significantly enhance vaccination coverage in remote areas and during mass immunization campaigns.

This study introduces a microneedle patch containing multiple microneedle arrays as a solution to the challenges of combination vaccines. Traditionally, five-in-one combination vaccines require mixing different vaccines, making selective administration difficult. Our five-in-one vaccine microneedle patch (five-in-one MN patch) allows for the simultaneous delivery of separate vaccines in a single application. The patch integrates four distinct vaccine microneedle arrays—Diphtheria and Tetanus combined, plus Pertussis, Hepatitis B, and *Haemophilus influenzae* type b—into one product. Each vaccine’s formulation was optimized individually, and the patch’s stability was confirmed over 12 months. Immunological efficacy was tested in animal models, demonstrating results comparable to traditional intramuscular injections. This innovative patch not only simplifies administration but also allows for easy removal or replacement of specific vaccines, addressing a significant limitation of current combination vaccines. Our findings suggest that the five-in-one MN patch is a promising alternative, enhancing vaccine accessibility and flexibility while maintaining stability and efficacy.

## 2. Materials and Methods

### 2.1. Measurement of Antigen Content

Thimerosal-free Diphtheria toxoid (DT), thimerosal-free Tetanus toxoid (TT), recombinant Hepatitis B surface antigen (HBsAg), thimerosal-free whole-cell Pertussis (wP), and Tetanus toxoid conjugated *Haemophilus influenzae* type b (Hib) vaccines were kindly provided by LG Chem (Seoul, Republic of Korea). Information about the pentavalent vaccine has been included in Supplementary Material S2.

The contents of the DT vaccine and the TT vaccine were determined by measuring their protein concentrations (PierceTM Detergent Compatible Bradford Assay Kit; Thermo Fisher Scientific (Waltham, MA, USA), cat. 23246) [32]. The protein contents of the HBsAg and Hib vaccine were measured by Modified Lowry protein assay (Thermo Fisher Scientific, cat. 23240) and Micro BCA protein assay (Thermo Fisher Scientific, cat. 23235) using BSA as a reference. The amount of wP vaccine was determined by opacity units (OUs) using the spectrophotometric method at 590 nm [33,34,35].

### 2.2. Analysis of Antigenicity of Vaccine

Indirect ELISA was performed for the DT vaccine, and sandwich ELISA was performed for the TT, HBsAg, and Hib vaccines. The detailed methods have been included in the Supplementary Materials. The antigenicity of the DT, TT, and Hib vaccines was analyzed by an in-house method with high specificity. The antigenic titer of HBsAg was determined using a sandwich ELISA-based commercial kit and followed the manufacturer’s instructions (Cat. No. 4110, Alpha Diagnostic International, Inc., San Antonio, TX, USA). An agglutinogen reaction test was performed for Pertussis [35]. The antigenicity of the standard curve and test sample was calculated by four-parameter nonlinear (4PL) regression. A statistical test was performed at the 0.01 level of the one-tailed test.

### 2.3. Fabrication of Vaccine Microneedle Array of DT-TT, wP, Hib, and HBsAg Vaccines

The target amount of each antigen was determined based on the content of commercial pediatric vaccine, which contains 15 Lf of Diphtheria toxoid, 10 Lf of Tetanus toxoid, 10 μg of Hepatitis B surface antigen, ≥4 IU of Pertussis antigen, and 30~50 μg of *Haemophilus influenzae* type b–Tetanus toxoid conjugate. Each antigen was loaded on a solid microneedle array by the dip-coating process described previously [36]. The composition of each coating solution and the number of coating cycles were adjusted to achieve the target dose of each vaccine.

As a first step of preparing a coating solution, each vaccine stock solution was concentrated to facilitate the dip-coating process. The DT and TT vaccines were concentrated using a centrifugal filter (10 kDa MWCO, 0.5 mL sample volume, Millipore, MA, USA) by centrifugation at 12,000× *g* for 5 min. The HBsAg and Hib vaccines were concentrated using a Vivaspin 20 centrifugal concentrator (10 kDa MWCO, 15 mL sample volume, Sartorius, Göttingen, Germany) by centrifugation at 3500× *g* for 20 min. For wP vaccine concentration, 1 mL of wP stock solution was put in a 1.7 mL microtube and concentrated by centrifugal precipitation at 5000× *g* for 5 min. The supernatant was removed after centrifugation, and a concentrated solution was prepared with the precipitated wP.

The excipients used in this study—α,α-Trehalose dihydrate (Cat # 90210, ≥99.0%, suitable for microbiology, composed of two α-glucose units, that is used as a protectant, stabilizer, and to support proper folding), carboxymethyl cellulose sodium salt (Cat # C5678, Sigma-Aldrich, MA, USA), and sodium hyaluronate (Cat # S0780000, Sigma-Aldrich)—were obtained from Sigma-Aldrich with analytical-grade purity, suitable for research purposes. Coating solutions for each vaccine were prepared in phosphate-buffered saline (PBS, pH 7.4, Thermo Fisher Scientific, Waltham, MA, USA) and are summarized in Table 1.

The master structure of microneedle arrays was manufactured by micro-milling. Micro-milling was employed to fabricate microneedles from a solid polymer block. A high-speed CNC micro-milling machine equipped with a tungsten carbide end mill (2MEM 008 016 S03, JJ Tools, Seoul, Republic of Korea) was used to carve the microneedle structures. The design parameters, including tip angle, base diameter, and height, were optimized using CAD modeling to ensure effective skin penetration while maintaining structural integrity. Machining was conducted at a spindle speed of 5000 rpm with a feed rate of 0.5 mm/min to minimize surface roughness. Post-milling, the microneedles were polished to remove debris and further improve tip sharpness. The microneedle array consisted of 97 obelisk-shaped microneedles (height: 800 μm, width: 370 μm) [36,37]. From the master structure, polymeric microneedle arrays were replicated by micro-molding. Polylactic acid (PLA; DURECT Corp., Birmingham, AL, USA) was used for the molding process. The contact angle was measured using a contact angle-meter (Femtobiomed, SmartDrop Plus, Seoul, Republic of Korea) with water as the test liquid. The prepared vaccine coating solution (Table 1) was dip-coated on the surface of the microneedles using the house-built coating apparatus. Viscosity was measured using a viscometer, and details about the viscometer were recorded (DV2TRCJ1, Brookfield, MA, USA). The coating parameters used in this study were a 350 μm dipping depth, a 10 mm/s downward speed, a 2 s immersion, and a 100 mm/s upward speed. The C-MNs were vacuum-dried (5 × 10^−4^ Torr) under ambient conditions (approximately 24 °C, 40% relative humidity) [36].

### 2.4. Stability Tests of Combination Vaccines for Each Vaccine

Long-term stability tests were conducted to observe the stability of each vaccine in the 5-in-1 vaccine microneedle patch. Microneedles prepared with each vaccine were stored at approximately 23 ± 3 °C and 40% relative humidity for 12 months using a temperature- and humidity-controlled chamber (Caron, 7000-75-2, Hustech, Seoul, Republic of Korea). Each vaccine was analyzed at time points of 0, 1, 2, 3, 6, 9, and 12 months after manufacture.

### 2.5. Vaccination in Animal Models

#### 2.5.1. Mouse Study: Evaluation of Immunogenicity

##### Effect of Diphtheria Toxoid and Tetanus Toxoid Vaccine Combination MN Array in Mouse

In order to examine the effect of the toxoid vaccine combination on immune responses, either Diphtheria toxoid and Tetanus toxoid vaccine combination or individual vaccines were administered to mice. Six-week-old female BALB/c mice (Orient Bio, Seongnam, Republic of Korea) were maintained under specific pathogen-free conditions in the experimental facility at the Yonsei University College of Medicine (Seoul, Republic of Korea), where they received sterilized food and water ad libitum. All animal experiments described were approved by the Institutional Animal Care and Use Committee of the Yonsei University College of Medicine (IACUC #2020-0222). Prior to microneedle vaccination, the hair on the dorsal skin of the mice was removed using a depilatory cream (Nair^TM^; Church and Dwight, Trenton, NJ, USA). Five mice per group were subsequently treated with either DT-TT vaccine combination C-MN or individually C-MNs. Mice received 1/10 of a human dose (1.5 Lf for Diphtheria toxoid vaccine and 1 Lf for Tetanus toxoid vaccine) using microneedles. The microneedles were applied to the hair-free region by thumb pressure and were further affixed to the skin for 30 min. For comparison, the same dose was also injected into mice intramuscularly with an injection volume of 50 μL. Serum samples were collected from the retro-orbital plexus 3, 6, and 9 weeks after immunization.

##### Efficacy of 5-in-1 Vaccine Microneedle Patch in Mouse

**Figure 1:**
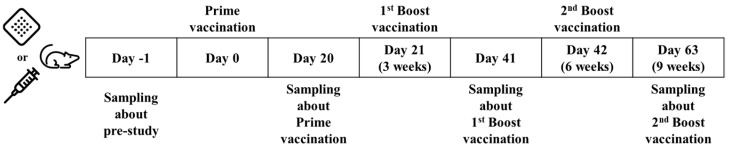
Timeline for vaccination and blood collection in mice: the timeline includes the use of either microneedle patches or traditional injection for vaccinations.

The efficacy of the 5-in-1 MN patch was examined in mice. This patch was prepared by combining four microneedle arrays (DT/TT, HBsAg, wP, and Hib vaccine). A commercial 5-in-1 vaccine, was injected intramuscularly as a control. Five-week-old female BALB/c mice were immunized three times on days 0, 21, and 42 with one-fifth of a human dose (Test number: SN22027) (Figure 1), with 10 mice per group, and all mice were raised in an M-cage (200 W × 260 L × 130 H [mm]) at 23 ± 3 °C/50 ± 20% (RH). Prior to vaccination, mice in the microneedle group were subjected to hair removal to facilitate microneedle administration. Hair on the back was first partly removed using a clipper (A6 Comfort, Oster, FL, USA) under anesthesia and then completely removed by applying about 1 mL of depilatory cream (Nicrin, Ildong Pharmaceutical Co., Ltd., Seoul, Republic of Korea), followed by cleaning three times with a water-soaked gauze pad for 3 min. Before hair removal and microneedle inoculation, the mice were anesthetized by injection with 300 μL of 2,2,2-Tribromoethanol (Avertin, Sigma-Aldrich, St. Louis, MI, USA). The microneedle array was administered on the backs of the mice by thumb pressure and remained attached for 30 min using a bandage. Intramuscular injection was performed in both thigh muscles (50 μL per thigh). Blood samples were obtained by retro-orbital bleeding on days-1, 20, 41, and 63 (Figure 1). The volume of blood extracted was 300 µL.

#### 2.5.2. Minipig Study: Evaluation of Immunogenicity

**Figure 2:**
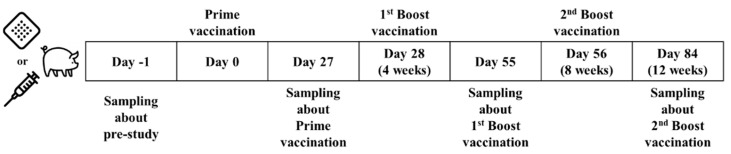
Schedule of vaccination and blood collection in minipigs: the timeline includes the use of either microneedle patches or traditional injection for vaccinations.

Female minipigs 3–4 months old (15–20 kg, Jeju Native Black Pig, CRONEX, Soeng-nam, Republic of Korea) were immunized with a human dose by either a 5-in-1 MN patch or intramuscular injection of a commercial vaccine after environmental adaptation. Similarly to the mouse study, hair from the backs of the minipigs in the microneedle group was gently removed the day before vaccine administration. The microneedle patch was applied to the hairless area and the minipigs were inoculated for 30 min. They received three doses, each 4 weeks apart, to allow for sufficient time for the antibody response to develop in minipigs and to match the administration schedule of the licensed vaccine. Blood was collected at 0, 4, 8, and 12 weeks after prime (the first vaccination), and serum samples were obtained after clotting and stored at −20 °C until analysis (Figure 2).

### 2.6. Analysis of Antigen-Specific Antibody Responses

#### Analysis of Antibody Titer

The antibody titer analyses were measured by ELISA. For mice, in-house ELISAs were used for the DT, TT, and HBsAg vaccines, and commercial ELISA kits (Alpha Diagnostic, Inc., San Antonio, TX, USA) were used for the wP (product #: 960-120-PHG) and Hib (product #: 980-120-PMG) vaccines. For minipigs, in-house ELISAs were used for analysis. The in-house ELISA showed high specificity with minimal background signal, and intra- and inter-assay variability remained below 10%, ensuring robust reproducibility.

To prepare ELISAs for antibody titer analysis, antigens were dispensed in a 96-well plate (Nunc Immuno Plate MaxiSorp, Thermo Fisher Scientific, Waltham, MA, USA) and incubated overnight at 4 °C. Concentrations of each antigen used are shown in Table 2, and 100 μL of antigen solution was dispensed per well.

After incubation, the plate was washed three times with a PBST followed by dispensing 200 μL of a blocking solution (5% skim milk for minipig ELISA, and 0.2% skim milk in PBS with 0.05% Tween 20 for mouse ELISA) in each well. The plate was then incubated at 37 °C for 1 h and washed three times. Serum samples were serially diluted with a blocking buffer to the desired concentrations, and 100 μL of the diluted samples were dispensed in each well followed by incubation at 37 °C for 2 h. After washing the plate five times, 100 μL of goat anti-Pig Fc IgG-HRP conjugate diluted 20,000 times with the blocking buffer was dispensed into each well and incubated at 37 °C for 1 h for minipig ELISA. For mouse ELISA, 100 μL of goat anti-Mouse IgG-HRP conjugate diluted 3000 times with the blocking buffer was dispensed. After incubation, the plate was washed five times, and 100 μL of TMB solution was dispensed into each well. The plate was then left in the dark for 15 min at room temperature for reaction, followed by dispensing 100 μL of 1 N sulfuric acid into each well to stop the reaction. The absorbance was measured using a microplate reader (Multiskan SkyHigh, Thermo Fisher Scientific, Waltham, MA, USA) at 450 nm as the measurement wavelength and 620–650 nm as the auxiliary wavelength. The point higher than the average background O.D value was defined as the endpoint, and a relative log titer was quantified.

### 2.7. Statistical Analysis

To determine statistical significance, a two-tailed Student’s *t* test was performed for comparing two different conditions, and one-way ANOVA was used for comparing multiple groups. Values of *p* < 0.05 were considered statistically significant.

## 3. Results and Discussion

### 3.1. Characteristics of Five-in-One Vaccine Microneedle Patch

#### 3.1.1. Physical Properties of Five-in-One Vaccine Microneedle Patch

**Figure 3:**
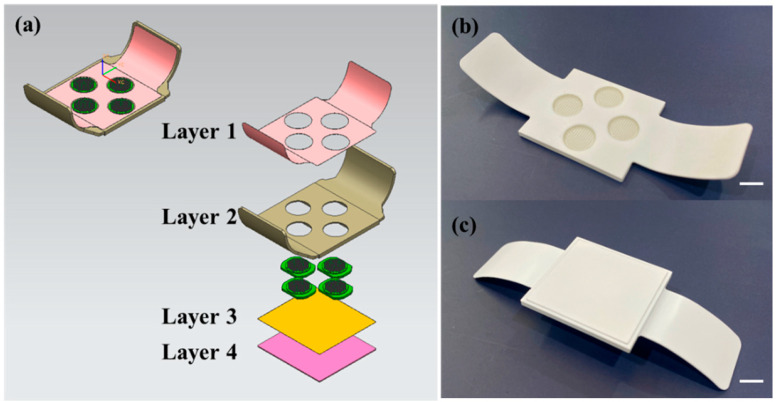
Concept of 5-in-1 MN patch assembled with four microneedle arrays and four layers: a microneedle patch that integrates combination vaccines into a single application, consisting of four coated microneedle (C-MN) arrays, each coated with different vaccines. (**a**) The C-MN housing consists of four layers: (1) upper adhesive layer, (2) array-holder layer, (3) lower adhesive layer, and (4) mechanical support layer. (**b,c**) Fabrication of the patch with four C-MN arrays (front and back). Scale bar represents 1 cm.

A five-in-one vaccine microneedle patch was prepared by integrating four microneedle arrays into one patch (Figure 3). Each microneedle array contained 97 obelisk-shaped microneedles (800 μm height, 370 μm width) on a 1 cm diameter circular base. The four arrays contained DT/TT, HBsAg, wP, and Hib vaccine, respectively. The composition and amount of coating formulation varied depending on vaccine type. To achieve high delivery efficiency, coating formulations were applied to the top portion of the microneedles.

The patch had four holes to hold the four microneedle arrays, designed to be applied to the wrist. The patch consisted of four layers. Layer 1, the upper part of the housing in contact with the skin, was an adhesive layer that fixed the patch on the skin. Layer 2 contained holes to hold arrays. Layer 3 was another adhesive layer for fixing the microneedle arrays in the holes of the patch. Layer 4 acted as a mechanical support, like a backing layer (Figure 3a).

**Figure 4:**
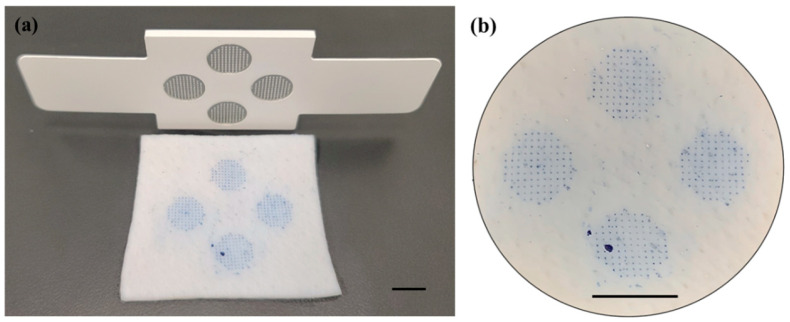
(**a**) Stained porcine tissue after 5-in-1 MN patch insertion showing that all microneedle arrays (four C-MN arrays) were successfully inserted into the porcine skin. (**b**) Enlarged image of treated skin showing successful penetration of all microneedles, forming blue dots. Scale bar represents 1 cm.

When the patch was attached to porcine skin and pressed evenly over the microneedle array with a pressure of 1 kg/cm^2^, each microneedle array successfully punctured the skin as shown in Figure 4a,b. Although the four microneedle arrays were completely inserted into the pigs’ skin in well-controlled environments, it could be challenging to successfully insert the arrays in clinical practice because pressure should be evenly applied over the large area of the patch. To confirm the successful administration of the microneedle patch, integrating feedback mechanisms such as color-changing pressure paper with the patch would be desirable.

#### 3.1.2. Formulation of Coating Solutions

The coating solution consisted of vaccine (s) and excipients that controlled the loading amount and stabilized antigen (s) during solidification and storage. The composition of the coating formulation was determined considering three factors: (1) compatibility with the manufacturing process, (2) the immunological stability of the dried vaccine, and (3) the dispersion stability of the dried vaccine.

For compatibility with the manufacturing process, the surface properties of the microneedles and the viscosity of the coating solution are important parameters. The flowability of the coating solution on the surface of the microneedles (PLA in this study) needs to be considered because a predetermined amount of antigen should be uniformly coated on the microneedle surface. In order to load the desired amount accurately, the tips of the microneedles must be evenly coated, which determines the amount of loading. The coating area is determined by the flowability of a coating solution, and the factors determining flowability are the interfacial tension between the coating solution and the microneedle and the viscosity of the coating solution. In this study, plasma treatment of the microneedles was also performed to increase the moistness of the coating solution on the microneedle surface. The contact angle of the coating solution on the PLA surface ranged between 50 and 60 degrees.

The viscosity of the coating solution was calculated to be between 100 and 300 cPs. CMC and HA were chosen as viscosity enhancers because they have been widely used as excipients for various vaccine microneedles and have shown minimal negative effect on antigenicity [38,39].

**Figure 5:**
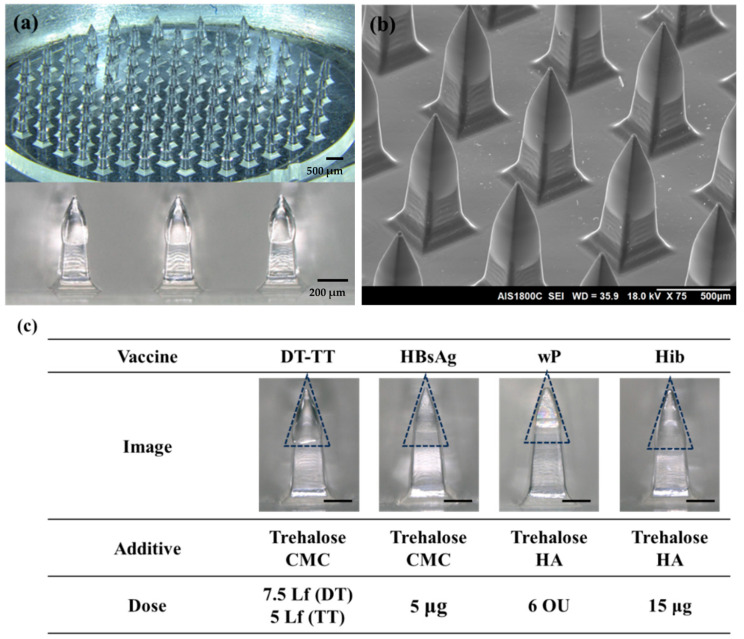
(**a**) Optical micrographs of vaccine C-MNs showing the overall structure and individual microneedle. (**b**) SEM image of vaccine C-MNs providing detailed visualization of microneedle tips. (**c**) Optical microscope images and doses of different vaccine C-MNs (vaccine additives and doses are specified). Scale bar represents 200 μm. The dashed line represents the cross-section of the coated solid formulation.

The suitability of the manufacturing process was confirmed by observing the solidified form (coating height, sharpness) of the formulation coated on the microneedle (Figure 5a–c). The criteria for the manufacturing process were the coating thickness, the geometry of the coated formulation, and the sharpness of the tips. When the height of the microneedle to be coated is divided by the depth of the coating well, the quotient is set to be between 1 and 1.2. At the same time, the exposed angle of the tip is kept sharp for successful insertion. The solidified formulation was positioned within half of the entire microneedle.

Excipients were chosen considering the immunological stability and dispersion stability of the antigens. DT, TT, and HBsAg are purified proteins, and microneedles containing those antigens have demonstrated improved stability during manufacturing and storage and immunological stability when disaccharides are used as a stabilizer [38,39,40]. Therefore, based on previous studies, trehalose, a disaccharide with a fast dissolution rate, was used as a stabilizer for DT, TT, and HBsAg.

The preparation of the Pertussis vaccine (wP)- and Hib vaccine-loaded microneedles and their stability are reported for the first time in this study. The formulation research for these two vaccines as microneedles was unprecedented, and especially for Hib and wP, the unique characteristics of these vaccines required different strategies to maintain antigenicity. The Pertussis vaccine is an inactivated bacterium of a whole cell, and to our knowledge, no microneedle study with the whole-cell pertussis (wP) vaccine has been reported. Since the wP vaccine is a suspension with a size of 600 nm, dispersion stability should be considered during redispersion in body fluid. Trehalose was first tested but did not improve the dispersion stability of wP particles. Next, CMC was added to the coating solution, but did not improve the dispersion of the solidified wP when reconstituted in a buffer solution; aggregated wP particles were observed in the solution. However, wP showed good dispersibility when HA was added in the formulation. Hib is a polysaccharide vaccine, but no microneedle studies with Hib vaccine have been reported. Similarly to wP, HA showed better dispersion stability of Hib compared to trehalose and CMC.

Figure 5 shows fabricated microneedle arrays coated with different antigens. The loading amount was determined based on 10 replicates, showing a mass variation within 10%. Close-up optical images of individual microneedles coated with the final coating formulation are shown in Figure 5c. Each microneedle array was loaded with half of an infant dose (7.5 Lf of DT, 5 Lf of TT, 5 μg of HBsAg, 6 OU of wP, and 15 μg of Hib), and the amount of the solid formulation varies depending on the vaccine. Each formulation was uniformly coated on the top half of the microneedles while pointy tips were maintained.

### 3.2. Long-Term Stability Test

**Figure 6:**
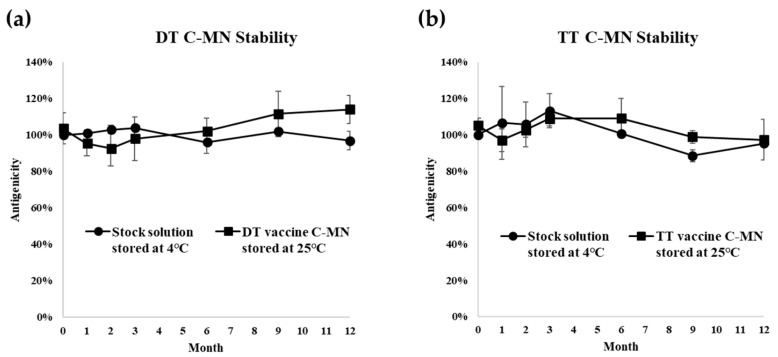

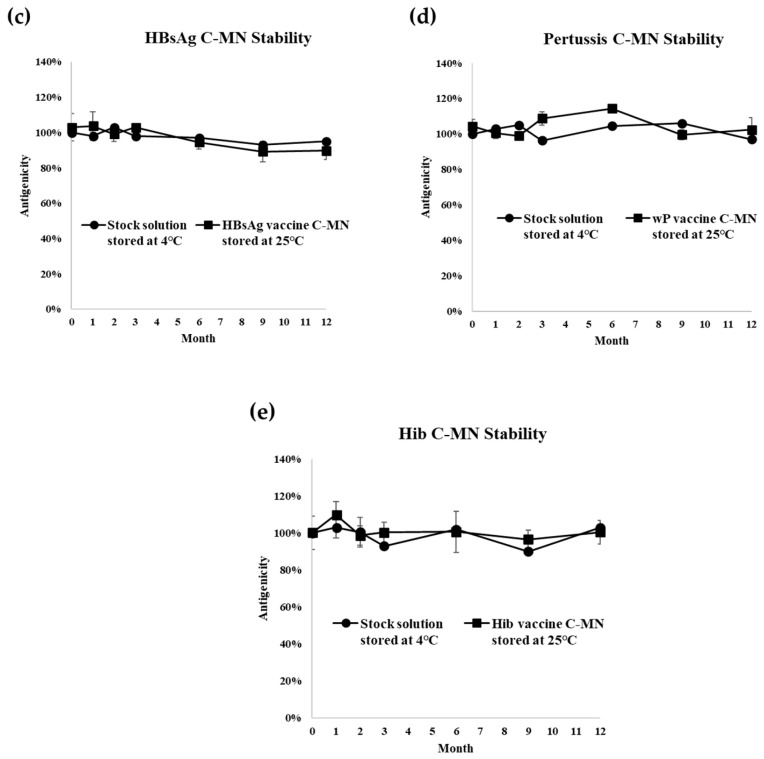
Long-term storage stability of each vaccine microneedle array for 12 months. (**a**) DT vaccine C-MN stability. (**b**) TT vaccine C-MN stability. (**c**) HBsAg vaccine C-MN stability. (**d**) wP vaccine C-MN stability. (**e**) Hib vaccine C-MN stability. Circles in the figure represent the antigenicity of the stock solution stored at 4 °C over time.

As shown in Figure 6, when microneedles loaded with each antigen are stored at room temperature for 12 months, the antigenicity of all vaccines was between 80% and 100% compared to the stock solutions stored at 4 °C. The DT, TT, and HBsAg vaccine microneedles were stable for a year at room temperature (Figure 6a–c). As reported in other studies of DT, TT, and HBsAg microneedles, these vaccines were stable for 1 year at room temperature by adding trehalose [38,39,40]. In this study, trehalose and CMC improved the storage stability of DT, TT, and HBsAg by fixing the antigen structure in a solid formulation. HA was selected as the dispersion stabilizer for wP vaccine microneedles. The addition of trehalose and HA showed that the wP vaccine can remain stable when stored for a year (Figure 6d). Hib vaccine microneedles also showed no change in stability during storage for a year with the addition of HA and trehalose (Figure 6e). For the wP and Hib vaccines, there was a dispersion problem of aggregation during redistribution without HA. As a result, for wP and Hib vaccines, the formulation based on HA and trehalose together maintains dispersion stability and immunological stability.

It is important to measure antigenicity to confirm the storage stability of the vaccine. Because the five-in-one vaccine microneedle patch eliminates inaccuracies in measurement and analysis caused by interactive interference between vaccines, antigenicity analysis can be performed accurately. It should be noted that wP and Hib vaccine-loaded microneedles and their stability are reported for the first time in this study; these vaccine microneedles demonstrated good long-term stability. Our results show that the antigenicity of combination vaccines solidified on microneedles can be maintained for one year at room temperature with proper excipients.

The five-in-one MN patch has the potential to enhance vaccine accessibility and distribution, particularly in low- and middle-income countries, by improving storage stability. This technology represents a significant advancement in vaccine delivery, contributing to global health equity and vaccine efficacy. Further studies should explore its application to other combination vaccines. This approach holds potential for significantly improving vaccination coverage worldwide.

Our stability study, while providing preliminary insights, has certain limitations, including the absence of accelerated stability testing and freeze–thaw cycle evaluations. Future investigations will address these gaps by incorporating stability testing under WHO-recommended conditions (40 °C/75% relative humidity) and freeze–thaw cycle assessments (3–5 cycles) to simulate real-world storage and handling scenarios. These additional studies will enhance the reliability of our findings and support broader clinical and regulatory applications.

### 3.3. Animal Study: Comprehensive Evaluation of Vaccine Efficacy and Immunogenicity in Mice and Pigs

#### 3.3.1. Antibody Response of DT-TT Vaccine C-MN Array in Mice

In order to prepare a combination vaccine in four microneedle arrays, DT and TT were mixed and co-administered on a single microneedle array due to their similar characteristics. For DT and TT vaccines, the same formulation can be used because the storage stabilizer of two vaccine microneedles is the same. To ensure robust immune responses and establish compatibility with the microneedle delivery system, DT and TT were first evaluated individually as separate MAP formulations before testing their combination in the five-in-one MAP. An immunogenicity test of DT-TT vaccine microneedles was performed in mice to observe the reduction in immunogenicity by interference of the vaccine.

**Figure 7:**
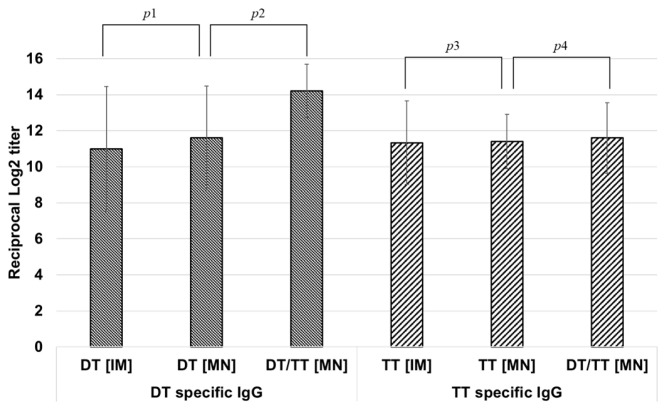
Comparison of DT- and TT-specific IgG levels induced by single-antigen vaccine (IM, MN) and combination vaccine (MN) and the *p* values p1, p2, p3, and p4. Statistical significance between groups was determined by a *t* test (*p* < 0.05), and there was no statistically significant difference (p2 < 0.5).

All vaccines were inoculated on a 1/10 dose basis. As shown in Figure 7, antigen-specific IgG titers induced by DT/TT microneedles were similar to those induced by single-antigen vaccine microneedles and intramuscular injection. The *p* values (p1-p4 in Figure 7) were 799, 111, 962, and 861, respectively, confirming that there was no statistically significant difference in antigenicity between the DT/TT combination vaccine and a single-antigen vaccine.

Since there was no immunological interference when toxoid vaccines are mixed, the DT and TT vaccine can be loaded onto a microneedle array together. Also, because the quantitative analysis method for the DT and TT vaccine is the same, the loaded amount of DT-TT vaccine on the microneedle array can be determined by using a quantitative measurement of the DT or TT vaccines.

In this study, the application force for MAPs was approximated at 1 kg/cm^2^ based on preliminary tests using a digital scale to standardize manual application. This approach ensured consistency during animal experiments, though we recognize the limitations of relying on manual pressure estimation. Future work will focus on integrating precise pressure-measurement tools to enhance the accuracy and reproducibility of MAP application, particularly for larger-scale or clinical studies.

#### 3.3.2. Antibody Responses of Five-in-One MN Patch in Mice

**Figure 8:**
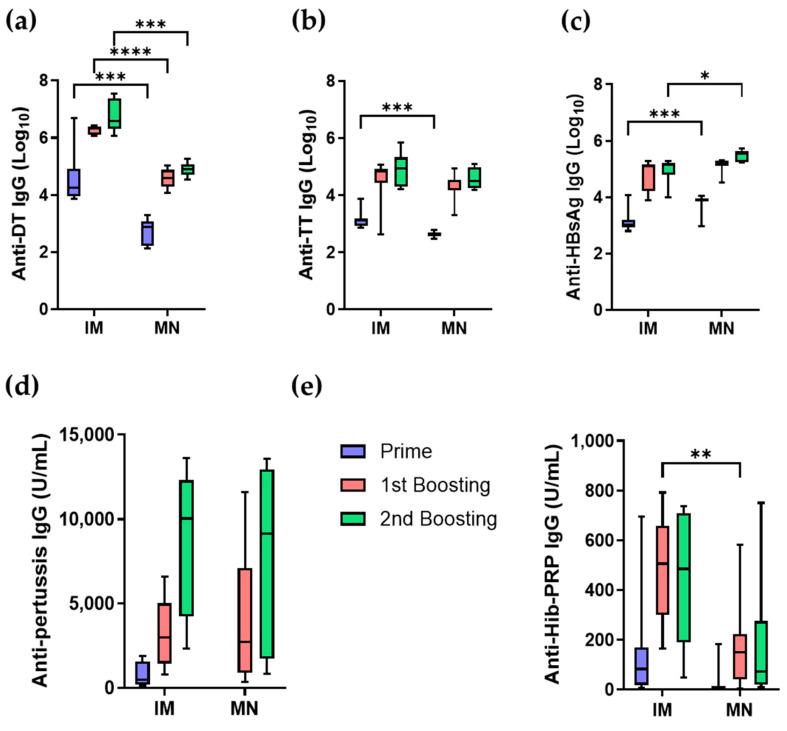
Comparison of antibody titers of 5-in-1 MN patch and pentavalent vaccine IM injection when mice were inoculated with a fifth of a human dose (* *p* < 0.1, ** *p* < 0.01, *** *p* < 0.001, **** *p* < 0.0001). (**a**) Anti-DT IgG, (**b**) anti-TT IgG, (**c**) anti-HBsAg IgG, (**d**) anti-Pertussis IgG, (**e**) anti-Hib-PRP IgG.

The efficacy of the five-in-one MN patch was examined in mice. Three doses (3 weeks apart) were administered using either microneedles or intramuscular injection. As shown in Figure 8, the DT-specific antibody titer induced by IM injection was higher than that induced by microneedles, whereas the HBsAg-specific antibody titer induced by microneedles was higher than that induced by IM. For TT, wP, and Hib vaccines, both methods showed similar antibody responses. Over the course of immunization, the DT-specific antibody titer gradually increased after three administrations of the microneedle (Figure 8a). The TT-specific antibody titer formed by the microneedle group was equivalent to the IM injection group, except for the prime vaccination (Figure 8b). For HBsAg, the microneedle group exhibited higher antibody titers after the first vaccination and the first boost (Figure 8c). The wP-specific antibody titer in the microneedle group was similar to the IM injection group, except for some differences in antibody levels (Figure 8d). Finally, for Hib, the microneedle group showed similar antibody titers to the IM injection group during the entire administration period (Figure 8e). These results suggest that antibody responses induced by microneedles are comparable to those induced by IM injection when the combination vaccine is administered to mice. The wide error bars in pertussis and Hib antibody levels reflect variability in microneedle patch adhesion and penetration, likely exacerbated by small animal-specific challenges such as residual fur and limited skin surface area, highlighting the need for optimized patch designs in preclinical studies.

#### 3.3.3. Antibody Responses of Five-in-One MN Patch in Minipigs

**Figure 9:**
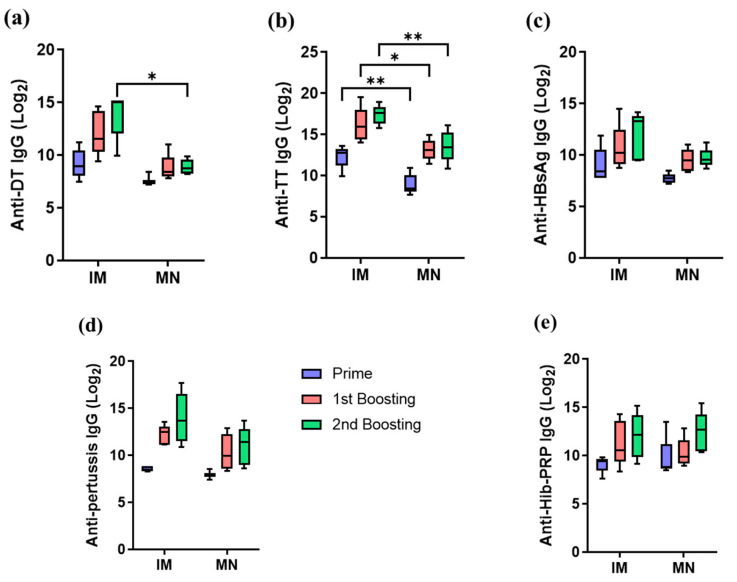
Comparison of antibody titers of 5-in-1 MN patch and pentavalent vaccine IM injection when minipigs were inoculated with human dose (* *p* < 0.1, ** *p* < 0.01). (**a**) Anti-DT IgG, (**b**) anti-TT IgG, (**c**) anti-HBsAg IgG, (**d**) anti-pertussis IgG, (**e**) anti-Hib-PRP IgG. Colors: blue (prime), red (1st boosting), green (2nd boosting).

Minipigs were vaccinated three times with a five-in-one MN patch, and antibody responses were compared with those induced by intramuscular injection. As shown in Figure 9, IM injection showed higher DT- and TT-specific antibody titers compared to the microneedle administration. For HBsAg, wP, and Hib vaccines, both groups showed similar antibody responses. It was observed that DT-specific antibody titers of the IM group were higher than those of the microneedle group after three vaccinations, but there was no statistical difference between the groups after the first injection and the first boost. Although microneedles induced lower DT-specific antibody responses compared to IM, the antibody level after three immunizations (antibody titer approximately 210) was sufficient for vaccine-specific immune response (Figure 9a). For Tetanus toxoid vaccination, IM injection showed higher antibody titers than those of microneedles throughout this study. However, in the microneedle group, the antibody titer of about 215 was formed after three administrations, indicating adequate vaccine-specific antibody formation (Figure 9b). For HBsAg, wP, and Hib, the microneedle group showed the same antibody titer as the IM group during the entire administration period, with no significant differences (Figure 9c–e). Notably, the five-in-one vaccine microneedles, administered without an adjuvant, produced immune responses comparable to those from the IM injection of pentavalent vaccine, which contains an adjuvant.

The immunological efficacy of the five-in-one MN patch was thoroughly evaluated in both mice and minipigs. Both models demonstrated that the five-in-one MN patch could administer multiple vaccines in one patch without mixing them, simplifying the administration process and maintaining the efficacy of each vaccine. Additionally, this innovative approach allows for the selective removal or replacement of specific vaccines, offering a flexible and effective alternative to traditional vaccination methods.

Moreover, the five-in-one MN patch has the potential to enhance vaccine accessibility and distribution, particularly in low- and middle-income countries, by improving storage stability and enabling self-administration. This technology represents a significant advancement in vaccine delivery, contributing to global health equity and vaccine efficacy. Further studies should evaluate the long-term stability of the patch at various temperatures and explore its application to other combination vaccines. This approach holds potential for significantly improving vaccination coverage worldwide. While adjuvants were excluded in this study to align with the current five-in-one vaccine formulation, future work should investigate skin-compatible adjuvants to enhance immune responses and enable dose sparing for MAP-based vaccination.

Immunogenicity studies were conducted in mice for dose-ranging and initial proof-of-concept, and in minipigs to assess human-like skin penetration and dose scalability, with the latter providing critical insights into the applicability of the microneedle system in human settings [41,42]. As shown in Figure 8 and Figure 9, the lower antibody titers observed with four microneedle arrays administered simultaneously indicate a reduction in delivery efficiency compared to single-array application. This reduction may be attributed to challenges in achieving uniform microneedle penetration when multiple arrays are applied concurrently. In the case of minipigs, residual hair or uneven skin surfaces likely interfered with consistent microneedle insertion, further lowering delivery efficiency and antibody responses. These results highlight the limitations of simultaneous multi-array administration and underscore the need for optimized application techniques to ensure consistent and efficient antigen delivery. Addressing these challenges will be critical for improving the performance of multi-array microneedle systems in preclinical and clinical settings. The large patch size and challenges in proper adhesion, particularly for young infants, represent a potential limitation, emphasizing the need for optimized application methods and verification techniques in future studies.

## 4. Conclusions

In this study, we developed and evaluated a five-in-one vaccine microneedle patch (five-in-one MN patch) that integrates four microneedle arrays for the delivery of Diphtheria, Tetanus, Pertussis, Hepatitis B, and *Haemophilus influenzae* type b vaccines. Each vaccine was individually formulated and coated onto separate microneedle arrays, which were then assembled into a single patch. The formulation of each vaccine was carefully designed to maintain antigen integrity during the manufacturing process and ensure storage stability.

Our findings demonstrated that the five-in-one MN patch maintained stable antigenicity for up to 12 months and showed immunological efficacy comparable to traditional intramuscular injections in both mice and minipigs. The patch not only simplifies the administration process, but also allows for easy removal or replacement of specific vaccines, providing a flexible and effective alternative to conventional vaccination methods.

This five-in-one vaccine microneedle patch can effectively deliver a combination vaccine as a single product. The microneedles containing whole-cell Pertussis and *Haemophilus influenzae* type b used in this study were manufactured for the first time. Our results showed that the combination vaccine patch (five-in-one MN patch) has immunological efficacy comparable to that of intramuscular injections of mixed vaccines. The five-in-one MN patch is a delivery method for administering various vaccines as a single product, and it can be used for various combination vaccines in the future.

The five-in-one MN patch represents a promising advancement in vaccine delivery technology, contributing to global health equity and vaccine efficacy. Further studies should evaluate the long-term stability of the patch at various temperatures and explore its application to other combination vaccines. This approach holds potential for significantly improving vaccination coverage worldwide.

## Figures and Tables

**Table 1:** Composition details of DT, TT, HBsAg, wP, and Hib vaccine coating solutions, including concentrations of vaccine and additive (trehalose, CMC, and HA).

Vaccine	Vaccine	Trehalose	CMC	HA
DT vaccine coating solution	1% (*w*/*v*)	15% (*w*/*v*)	1% (*w*/*v*)	-
TT vaccine coating solution	1% (*w*/*v*)	15% (*w*/*v*)	1% (*w*/*v*)	-
HBsAg vaccine coating solution	1% (*w*/*v*)	15% (*w*/*v*)	1% (*w*/*v*)	-
wP vaccine coating solution	4.5% (*w*/*v*)	1.5% (*w*/*v*)	-	1.5% (*w*/*v*)
Hib vaccine coating solution	1% (*w*/*v*)	15% (*w*/*v*)	-	1% (*w*/*v*)

**Table 2:** The concentration of various antigens used in experiments with mice and minipigs.

Antigen	Mouse	Minipig
Diphtheria toxoid	62.5 ng/mL	0.1 μg/mL
Tetanus toxoid	62.5 ng/mL	0.1 μg/mL
Hepatitis B surface antigen	2 μg/mL	1 μg/mL
Pertussis-PRN	N/A	0.25 μg/mL
Hib-PRP	N/A	2 μg/mL

## Data Availability

The data presented in this study are available on request from the corresponding author.

## Supplementary Materials

The following supporting information can be downloaded at: https://www.mdpi.com/article/10.3390/pharmaceutics16121631/s1, S1. Antigen Content Quantification. S2. Information of Pentavalent vaccine. Table S1. Comparison of Protein Assay Methods for Antigen Content Quantification: Methods, Ranges, and Standards. Table S2. Comparison of ELISA Assay Methods for Antigen Content Quantification: Methods, Ranges, and Standards. Table S3. Effect of additive on Redispersion Efficiency of Pertussis-Coated Microneedles.
