# Supplementary material for: Development and Evaluation of Five-in-One Vaccine Microneedle Array Patch for Diphtheria, Tetanus, Pertussis, Hepatitis B, and Haemophilus influenzae Type b: Immunological Efficacy and Long-Term Stability
Source: Pharmaceutics. 2024 Dec 23;16(12):1631. doi: 10.3390/pharmaceutics16121631 (PMC11677855; doi:10.3390/pharmaceutics16121631)
Supplement: Supplementary file 1 [file pharmaceutics-16-01631-s001.zip › pharmaceutics-3343588-supplementary.pdf]

Supplementary Material

S1. Antigen Content Quantification

Table S1. Comparison of Protein Assay Methods for Antigen Content Quantification: Methods, Ranges, and Standards

Antigen	Protein Quantification Method	Working Range	Recommended Range	Standard	Analysis Formula	Reference
**DT**	DC Bradford assay	100-1500 µg/mL,<br>2-25 µg/mL	400-600 µg/mL,<br>8-15 µg/mL	Albumin Standard Ampules, 2mg/mL	Polynomial (x: Dose, y: OD)	Analysis was performed according to the manufacturer's protocol instructions.
**TT**	DC Bradford assay	100-1500 µg/mL,<br>2-25 µg/mL	400-600 µg/mL,<br>8-15 µg/mL	Albumin Standard Ampules, 2mg/mL	Polynomial (x: Dose, y: OD)	Analysis was performed according to the manufacturer's protocol instructions.
**Pertussis**	Opacity unit	2.5-30 OU/mL	5-25 OU/mL	-	Linear (x: Dose, y: OD)	Study on Toxicity Reduction and Potency Induction in Whole-cell Pertussis Vaccine by Developing a New Optimal Inactivation Condition Processed on Bordetella pertussis
**HepB**	Lowry assay, Micro BCA	20-100 µg/mL,<br>2-40 µg/mL	40-60 µg/mL,<br>10-25 µg/mL	Albumin Standard Ampules, 2mg/mL	Linear/Polynomial (x: Dose, y: OD)	Analysis was performed according to the manufacturer's protocol instructions.
**Hib**	Lowry assay, Micro BCA	20-100 µg/mL,<br>2-40 µg/mL	40-60 µg/mL,<br>10-25 µg/mL	Albumin Standard Ampules, 2mg/mL	Linear (x: Dose, y: OD)	Analysis was performed according to the manufacturer's protocol instructions.

Table S2. Comparison of ELISA Assay Methods for Antigen Content Quantification: Methods, Ranges, and Standards

Antigen	ELISA for Antigenicity	Working Range	Analysis Formula	Reference
**DT**	Sandwich ELISA	0.0078-1 Lf/mL	Linear (x: $\log_2$ Dose, y: $\log_2$ OD)	We have conducted sufficient method optimization and validation.
**TT**	Sandwich	0.00006-0.008 Lf/mL	Linear	We have conducted sufficient

	ELISA		(x: $\log_2$ Dose, y: $\log_2$ OD)	method optimization and validation.
**Pertussis**	Agglutination	-	-	Manual of Quality Control of Diphtheria, Tetanus and Pertussis Vaccines
**HepB**	Sandwich ELISA	1.25-20 ng/mL	Linear<br>(x: ln Dose, y: ln OD)	Analysis was performed according to the manufacturer's protocol instructions.
**Hib**	Sandwich ELISA	0.23-14.5 μg/mL	Linear<br>(x: Dose, y: OD)	We have conducted sufficient method optimization and validation.

S1.1 Analysis of Antigenicity of DT Vaccine

The antigenicity of the DT vaccine was measured by an indirect ELISA. Sodium carbonate (Sigma-Aldrich Cat. No. S7759) and sodium bicarbonate (Sigma-Aldrich Cat. No. S5761) were dissolved in water to prepare the carbonate buffer solution. Diphtheria toxoid bulk (2000 Lf/mL) was diluted using the carbonate buffer to a concentration of 0.1 Lf/mL and further diluted two-fold. For antigen coating on plates, dispense 100 μL of DT bulk and sample into a plate (Costar Cat. No. 3590) and incubate overnight at 4°C. After washing 3 times with PBST (PBS + 0.05% Tween20), the plate was incubated for 90 min at 37°C with 2% skim milk in PBS and then washed 5 times with PBST. Anti-DT mouse IgG (1:2500 dilution; Abcam Cat. No. 53827) was dispensed at 100 μL per well and the plate was incubated for 60 min at 37°C and washed 5 times. 100 μL of anti-mouse IgG-HRP (1:2500 dilution; Southern Biotech Cat. No. 1030-05) was then dispensed per well. The plate was incubated for 30 min at 37°C and washed 5 times. 100 μL of TMB solution (Abcam Cat. No. 171527) was added to each well and incubated for 20 min at room temperature in the dark. The reaction was stopped by the addition of 100 μL of stop solution (Abcam Cat. No. 171529). For analysis, absorbance was measured at 450 nm using a microplate reader (FLUOstar Omega, BMG LABTECH).

S1.2 Analysis of Antigenicity of TT Vaccine

The antigenicity of the Tetanus toxoid was measured by a sandwich ELISA. Anti-tetanus serum (NCIPD) at 1890 IU/mL was diluted with carbonate buffer to a concentration of 0.08316 IU/mL and 100 μL of the diluted serum was dispensed into each well. The plate was incubated overnight at 4°C and washed 5 times. 300 μL of 2% skim milk in PBST was dispensed into each well and incubated for 90 min at 37°C. After washing, tetanus toxoid bulk (1100 Lf/mL) and the sample were diluted with 2% skim milk in PBST to a concentration of 0.008 Lf/mL and further diluted two-fold in the same manner, then incubated for 120 min at 37°C. After washing 5 times, 100 μL

of primary antibody (1:2500 dilution; anti-TT rabbit IgG, Abcam Cat. No. 53829) was dispensed per well and the plate was incubated for 60 min at 37°C and washed 5 times. The secondary antibody (1:2500 dilution; Goat anti-Rabbit IgG-HRP, Abcam Cat. No. 205718) was incubated with 100 μL per well for 30 min at 37°C and washed 5 times. 100 μL of TMB solution was added and incubated for 15 min at room temperature in the dark. The reaction was stopped by adding 100 μL of stop solution and analyzing with an absorbance meter at 450 nm.

S1.3 Analysis of Antigenicity of HBsAg Vaccine

The antigenic titer of the HBsAg vaccine was determined using a commercial sandwich ELISA kit (Alpha Diagnostic International Inc. Cat. No. 4110) following the manufacturer's instructions.

S1.4 Analysis of Antigenicity of Hib Vaccine

The antigenic titer of the Hib vaccine was determined by a sandwich ELISA, which identifies the PRP-TT conjugate of the Hib vaccine. Goat anti-Tetanus Toxoid (5 mg/mL, Bio-Rad Cat. No. 8750-2059G) was diluted 1000 times with a carbonate buffer and dispensed into wells (100 μL per well). The plate was incubated at 37°C for 2 hours. After washing 3 times with PBST, samples were diluted with a diluent (10, 50, and 100 times) according to the concentration of the stock solution. 100 μL of the diluted sample was dispensed into 8 wells, and 100 μL of the diluted sample into 8 wells as a blank. The plate was incubated for 3 hours at room temperature on a shaker, followed by washing 5 times. For detection, rabbit anti-PRP-HRP conjugate was diluted 2500 times with 1% normal rabbit serum. 100 μL of the diluted conjugate was dispensed into each well and the plate was incubated for 1 hour at room temperature on a shaker at 500 rpm. After washing the plate 5 times, 100 μL of TMB solution was added to each well and incubated for 20 min at room temperature in the dark. The reaction was stopped by adding 100 μL of a stop solution. For analysis, absorbance was measured at 450 nm using the microplate reader.

S1.5 Analysis of Antigenicity of wP Vaccine

The agglutinogen test of whole-cell Pertussis was carried out with Bordetella Pertussis anti-agglutinogen 1, 2, and 3 (NIBSC code: 89/596, 89/598, 89/600). 10 μL of each agglutinogen was dispensed on an individual slide glass. 10 μL of sterile water was dispensed on the other side of each slide glass as a negative control. 10 μL of

homogenized whole-cell pertussis test solution was then dispensed on each side of the slide glasses where agglutinogens and water were dispensed. The reaction was observed while gently shaking the slide glass. Antigenicity was determined by observing aggregation.

S2. Information of Pentavalent vaccine

The vaccine is Eupenta from LG Chem Ltd and has been approved by the WHO. The current prequalification status is valid and the effective date is 10/02/2016. The vaccine type is Diphtheria-Tetanus-Pertussis (whole cell)-Hepatitis B-Haemophilus influenzae type b. LG Chem announced that it has signed a contract worth a total of 200 million dollars (approximately 260 billion won) for the 5-valent (diphtheria, tetanus, pertussis, hepatitis B, meningitis) combination vaccine 'Eupenta' through a UNICEF bid. This contract is a contract to supply the 5-valent combination vaccine Eupenta for 100 million dollars over 5 years (2023-2027). Based on this contract, LG Chem will supply enough vaccines to prevent infectious diseases in approximately 80 million infants and young children around the world.

S3. Information of Additives

Table S3. Effect of additive on Redispersion Efficiency of Pertussis-Coated Microneedles

**Saple**	**Only** Pertussis-coated microneedles	Pertussis with **CMC** -coated microneedles	Pertussis with **trehalose** -coated microneedles	Pertussis with **HA** -coated microneedles
**Image**	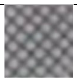	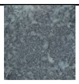	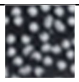	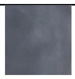
**Observation**	No redispersion	Partial redispersion	No redispersion	Successful redispersion

*CMC : carboxymethylcellulose, **HA : Hyaluronic acid
